# Equilibrium, Kinetic, and Thermodynamic Studies on the Adsorption of Cadmium from Aqueous Solution by Modified Biomass Ash

**DOI:** 10.1155/2017/3695604

**Published:** 2017-02-28

**Authors:** Lei Xu, Xuebo Zheng, Hongbiao Cui, Zhenqiu Zhu, Jiani Liang, Jing Zhou

**Affiliations:** ^1^Institute of Soil Science, Chinese Academy of Sciences, Nanjing 210008, China; ^2^Key Laboratory of Soil Environment and Pollution Remediation, Chinese Academy of Science, Nanjing 210008, China; ^3^National Engineering and Technology Research Center for Red Soil Improvement, Red Soil Ecological Experiment Station, Chinese Academy of Sciences, Liujiazhan Plantation, Yingtan 335211, China; ^4^University of Chinese Academy of Sciences, Beijing 100049, China; ^5^School of Earth and Environment, Anhui University of Science and Technology, Huainan 232001, China

## Abstract

Natural biomass ash of agricultural residuals was collected from a power plant and modified with hexagonal mesoporous silica and functionalized with 3-aminopropyltriethoxysilane. The physicochemical and morphological properties of the biomass ash were analyzed by ICP-OES, SEM, TEM-EDS, FTIR, and BET analysis. The adsorption behavior of the modified product for Cd^2+^ in aqueous solution was studied as a function of pH, initial metal concentration, equilibrium time, and temperature. Results showed that the specific surface area of the modified product was 9 times that of the natural biomass ash. The modified biomass ash exhibited high affinity for Cd^2+^ and its adsorption capacity increased sharply with increasing pH from 4.0 to 6.0. The maximum adsorption capacity was 23.95 mg/g in a pH 5 solution with an initial metal concentration of 50 mg/L and a contact time of 90 min. The adsorption of Cd^2+^ onto the modified biomass ash was well fitted to the Langmuir model and it followed pseudo-second-order kinetics. Thermodynamic analysis results showed that the adsorption of Cd^2+^ was spontaneous and endothermic in nature. The results suggest that the modified biomass ash is promising for use as an inexpensive and effective adsorbent for Cd^2+^ removal from aqueous solution.

## 1. Introduction

Biomass ash is a by-product from the combustion of biomass feedstock at a combustion plant. During combustion, the inorganic constituents of biomass form biomass ash, increasing its accumulation in the environment [1, 2]. Since biomass ash shows good adsorption capacity for heavy metals, it has been increasingly used as a low-cost and environment-friendly adsorbent to remove the heavy metals such as Cd^2+^, Pb^2+^, and Cu^2+^ from wastewater [3, 4]. Removal of heavy metals by adsorption is considered simpler to operate and less expensive, as compared with traditional techniques such as ion exchange, chemical precipitation, electrochemical treatment, membrane technology, evaporation, and solidification [5]. However, the adsorption capacity of natural biomass ash for particular heavy metals from aqueous solution is lower than some commercial or modified adsorbents [6]. Appropriate modification of biomass ash may enhance its adsorption capacity of heavy metals from wastewater [7].

Various mesoporous materials based on silicon dioxide have been extensively studied and partially commercialized. These materials are regarded as good adsorbents because of their large specific surface area, high thermal and mechanical stability, homogeneous pore morphology, high functionalization, and high adsorption capacity [8]. Furthermore, the adsorption capacity of mesoporous materials for heavy metal ions has been increased by combining particular organic functionalities on the surface and/or inside the pores of the material [9–11]. The modified materials with nanostructures have been shown to effectively remove heavy metals from industrial wastewater [12]. Some studies have been conducted to synthesize a new material by using coal fly ash and a functionalized mesoporous material. In fact, biomass ash has a high silicon content which gives it the potential to become a silica skeleton [13]. However, biomass ash has never been modified by this way.

Cadmium (Cd) is a highly toxic and carcinogenic heavy metal discharged into water from mining, smelting, electroplating, and alloy manufacturing, among various industries [14]. Unlike many organic pollutants, Cd cannot be degraded and constantly accumulate in the environment, causing a serious threat to aquatic ecosystems and human health [15–19]. It is therefore critical to remove Cd from industrial wastewater before discharge into the environment. Removal of Cd ions (Cd^2+^) from aqueous solutions by absorption has been reported, for example, using peanut shell, zeolite, coal fly ash, activated carbon, and biochar [20–23]. To our knowledge, the removal of Cd^2+^ by adsorption onto biomass ash, particularly its modified product, has never been studied.

The aim of this study was to develop a low-cost and efficient mesoporous adsorbent with high stability in acidic medium by modification of biomass ash with mesoporous silica and organosilane. The physicochemical and morphological properties of the modified product were characterized. The adsorption characteristics of this modified biomass ash for Cd^2+^ in aqueous solution had been researched using batch experiments. The results will contribute to understanding about the kinetic and thermodynamic mechanisms of this new material for removal of Cd^2+^ from aqueous solution.

## 2. Materials and Methods

### 2.1. Biomass Ash

The biomass ash sample was collected from a power plant firing agricultural residues in Anhui province, China. A mixture of agricultural residues, such as wheat stem, maize straw, groundnut shell, and cotton stalk, was used as feedstock in the power plant. The residues were fired in a traveling-grate furnace at ~850°C with excess air. The biomass ash was collected from the tank located below the grate.

### 2.2. Biomass Ash Modification

The biomass ash was modified with a hexagonal mesoporous silica (HMS) matrix by cocondensation [8]. First, 1.24 g of dodecylamine was dissolved in 10 mL of alcohol, followed by addition of a mixture of 1.24 g of biomass ash in 90 mL of ultrapure water (CN61 M-UPR-I-20L) under stirring at 1000 rpm. Next, 6.09 mL of tetraethyl orthosilicate and 0.71 mL of 10% (w/v) 3-aminopropyltriethoxysilane [APS, NH_2_(CH_2_)3Si (OC_2_H_5_)_3_], an organosilane, were added into the reaction mixture. After 30 s, 0.94 mL of trimethylbenzene was added and the mixture was then stirred for 24 h. Finally, the mixture was filtered through a 0.45 *μ*m filter membrane and the residue was air dried at room temperature. The remaining trimethylbenzene was Soxhlet extracted with 125 mL of alcohol for 5 h and the sample was air dried at room temperature for 24 h.

### 2.3. Physicochemical and Morphological Characterizations

The physicochemical and morphological properties of the natural and modified biomass ash samples were evaluated. The elemental composition was analyzed by inductively coupled plasma-optical emission (ICP-OES) spectrometry using a Perkin Elmer Optima 2000 DV system (Perkin Elmer, Waltham, MA, USA). The surface morphology was observed using an S-4800 scanning electron microscope (SEM; Hitachi, Tokyo, Japan) [24]. Topographic analysis and quantification of the components were performed using a JEM-2010HT transmission electron microscope (TEM; JEOL Ltd., Akishima, Tokyo, Japan) equipped with an energy-dispersive spectrometer (EDS). Functional groups were characterized by Fourier transform-infrared spectrometry (FTIR) using a Spectrum Two™ IR spectrometer (Perkin Elmer) in the range 4000–500 cm^−1^ [25]. The specific surface area of the materials was determined by BET method [24].

### 2.4. Adsorption Experiments

In order to evaluate the Cd^2+^ adsorption capacity of biomass ash and the modified material, batch adsorption experiments were carried out using guaranteed reagents. Cadmium solutions used in the experiment were prepared from a standard solution of 100 mg/L Cd(NO_3_)_2_ in 0.5 mol/L HNO_3_ and ultrapure water (CN61M-UPR-I-20L). The pH was adjusted with 0.1 mol/L HNO_3_ and NaOH.

To determine the effect of pH, 0.1 g of adsorbent was added to 25 mL of 50 mg/L Cd^2+^ solution in the pH range of 2.0–8.0. Adsorption isotherms were obtained with several initial adsorbate concentrations from 50 to 100 mg/L in 25 mL of solution at pH 5.0 with 0.1 g of adsorbent. The experiments were carried out in 50 mL centrifuge tubes at 30°C with shaking at 150 rpm for 24 h. For thermodynamic studies, the experiments were repeated at 45°C and 60°C. The adsorption kinetics was studied by adding 0.2 g of adsorbent into 100 mL of 100 mg/L Cd^2+^ solution at pH 5.0. The experiments were carried out on a 150 rpm reciprocal shaker at 30°C for varying time intervals, 0.5, 1, 2, 3, 5, 10, 15, 30, 60, 90, 120 180, 240, and 300 min.

The samples (5 mL each) were centrifuged at 4,000 rpm for 10 min. The supernatants were filtered with a 0.45 *μ*m membrane and the Cd^2+^ concentrations in the aqueous phase were determined by atomic absorption spectrophotometry (SpectrAA-220, Varian, Palo Alto, CA, USA).

### 2.5. Data Processing

Microsoft Excel 2010 (Microsoft Corp., Redmond, WA, USA) and SPSS 20.0 (IBM SPSS, Somers, NY, USA) were used to process the data.

## 3. Results and Discussion

### 3.1. Physicochemical and Morphological Characteristics of Natural and Modified Biomass Ash

**Figure 1:**
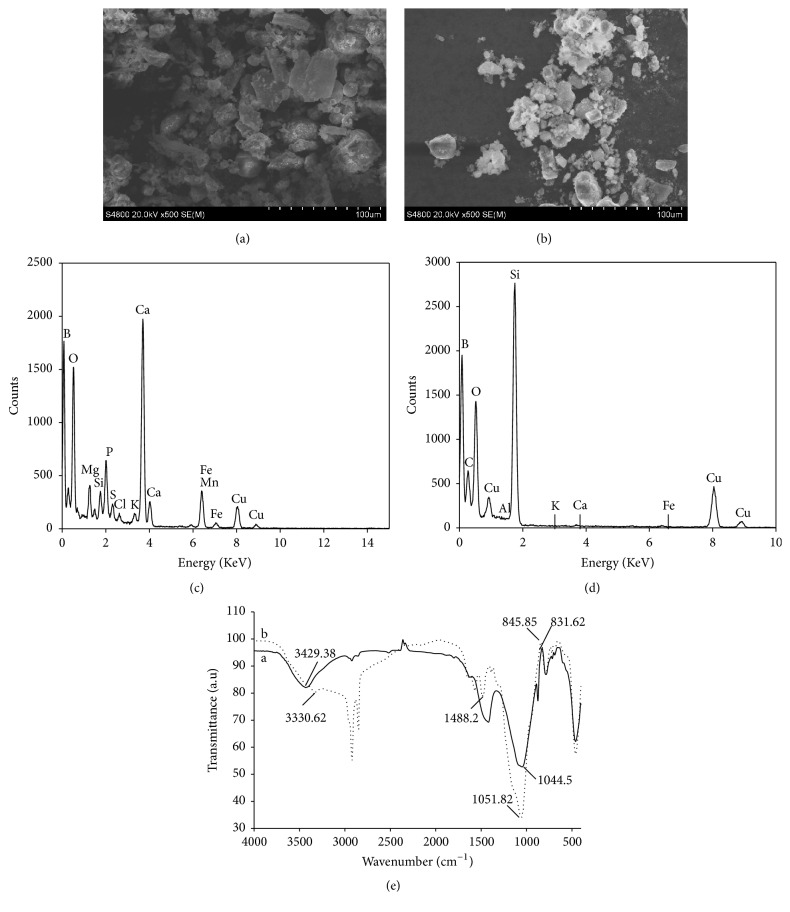
(a) SEM of biomass ash at 20 KeV; magnification 500. (b) SEM of synthesized matrix at 20 keV; magnification 500. (c) EDS analysis of elemental composition of biomass ash. (d) EDS analysis of elemental composition of modified biomass ash and (e) FT-IR spectra: (a) biomass ash and (b) synthesized matrix.

The elemental analysis results showed that the major elements present in the natural biomass ash were silicon, calcium, and potassium at concentrations of 120.40, 43.14, and 33.11 mg/g, respectively (Table 1). The SEM analysis results revealed the presence of numerous sheet particles with diameters between 10 and 60 *μ*m in the natural biomass ash; these particles were dispersed well (Figure 1(a)). The appearance of the modified biomass ash was markedly different from the natural biomass ash with a clear spherical structure. The hexagonal structure changed after reaction with biomass ash. In the presence of silicate, the modified hexagonal structure acted as a catalyst, increasing the reactivity of tetraethyl orthosilicate. The specific surface area of the modified biomass ash improved, and the surface was more smooth and homogeneous (Figure 1(b)).

The TEM-EDS results of elemental composition showed the presence of C, O, Si, Al, Fe, and K in the modified biomass ash (Figure 1(d)). FTIR analysis confirmed the presence of N in the modified biomass ash, indicating that the biomass ash had been functionalized by the primary amine (APS). The FTIR spectrum of the modified biomass ash (Figure 1(e)) demonstrates an intense absorption band at 3330 cm^−1^; this can be attributed to the O–H bonds of the silanol groups. Other absorption bands appear at 850 and 1044 cm^−1^, which was related to the symmetric and asymmetric Si–O–Si vibrations, respectively. After the functionalization with 10% APS and HMS matrix, the spectrum of the modified biomass ash was clearly different compared with that of the natural biomass ash; a broad signal appears between 3000 and 3600 cm^−1^ which can be attributed to the improved number of silanol groups [24]. The stretching bands can be attributed to the N–H group of APS, and the band at 1488.2 cm^−1^ corresponds to the bending vibration of the N–H groups.

The results of BET analysis showed that the specific surface area of modified biomass ash was 9 times that of the nature biomass ash, 21.38 ± 0.17 m^2^/g versus 185.81 ± 0.15 m^2^/g (Table 2). The improved specific surface area indicates that a functional mesoporous material was obtained.

### 3.2. Effect of pH on Cadmium Adsorption Capacity

**Figure 2:**
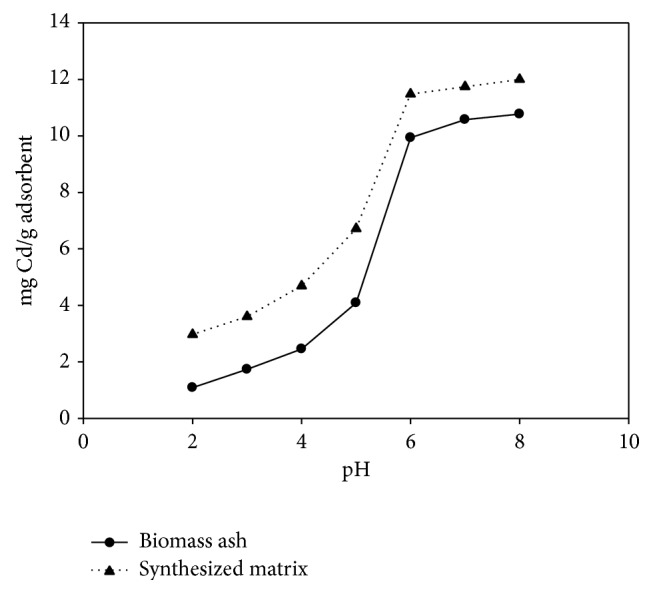
Effect of pH on the adsorption of Cd^2+^ on biomass ash and modified biomass ash (initial concentrations of Cd^2+^, 50 mg/L; biomass ash concentration, 4 g/L;* T* = 30°C).

The pH is an important factor in removing heavy metals by adsorption from aqueous solutions [26, 27].  Figure 2 shows the effects of different initial pH values on the load capacity (mg/g) for Cd^2+^ of the two materials. The Cd^2+^ adsorption was found to be highly pH-dependent. At pH < 4, both the natural and modified biomass ash showed a significantly low adsorption capacity, for natural biomass ash, the adsorption capacity was only 1 mg/g, and as to the modified biomass ash, it was about three times larger than the natural biomass ash. When the pH of the solution was increased, the adsorption capacity of the biomass ash and the modified biomass ash increased sharply, and when the pH reached 6, the adsorption capacity of modified biomass ash reached 12 mg/g which was 4 times more than the pH 2. The effect of pH on the adsorption of metals can be attributed to the surface charge of the adsorbent and the distribution of metal species [28].

The surface of biomass ash has a negative charge, which is transient and depends on the pH of the solution [28]. When the pH in solution is substantially low, the number of H_3_O^+^ exceeds that of metal ions by many times; thus, the adsorbent surface is almost completely covered by H_3_O^+^, leading to a lower adsorption capacity for metal ions [29]. When the pH is gradually increased, an increasing number of H_3_O^+^ are removed from the adsorbent surface, allowing the metal ions to approach to the active adsorption sites; this increases the binding of metal ions to the modified adsorbent surface through the mechanism of ion exchange [29]. At pH > 4, the H_3_O^+^ concentration is markedly reduced, which is beneficial to the adsorption of metal ions on the surface of the adsorbent. This phenomenon can be attributed to the existence of oxides such as SiO_2_, Fe_2_O_3_, and Al_2_O_3_, whose surface charge depends mainly on the pH in the solution. The exchange mechanism of H^+^ and metal ions in solution can be represented by the following equations:

(1) XOH+H3O+⟶XOH2++H2O

(2) XOH+OH−⟶XO−+H2O

(3) 2XO−+M2+⟶XO2M,

where X represents Si, Fe, and Al; M represents metal.

With further increase of pH, the negative charge on the adsorbent surface is improved, thus increasing the electrostatic force between the adsorbent and adsorbate [30, 31]. The maximum adsorption efficiency of Cd^2+^ on the natural and modified biomass ash was observed between pH 5 and pH 6. At pH > 6, the weak adsorption of Cd^2+^ can be attributed to the precipitation of Cd species such as carbonates or hydroxides (Figure 2), according to the distribution of metal species affected by pH [32].

The modified biomass ash was functionalized with NH_2_ groups, forming an amino-Cd complex with a higher stability constant favorable for the formation of this compound. The stability of the compound mainly depends on the pH which must be near 7 [8]. At pH < 4, the H^+^ ions react with the lone pair of electrons of N, preventing the binding of Cd^2+^ with NH_2_ groups; at pH > 6, the Cd^2+^ ions precipitate due to the formation of insoluble species.

### 3.3. Adsorption Isotherms

Table 3 compares the Cd^2+^ adsorption capacity of the modified biomass ash with several types of adsorbents reported at the pH of 5-6. The modified biomass ash appears superior to most of the other materials, especially peanut shell, banana peel, sepiolite, zeolite, and natural biomass ash.

The Langmuir and Freundlich adsorption models were used to fit the adsorption data of the nature biomass ash and modified biomass ash to Cd^2+^ in aqueous solution.

The Langmuir isotherm is usually applied to monolayer adsorption on homogeneous surfaces with a finite number of adsorption sites. The linear form of Langmuir equation can be expressed as follows [33]:

(4) $$\begin{array}{c} \frac{C_{e}}{q_{e}}=\frac{1}{q_{L}\cdot K_{L}}+\frac{C_{e}}{q_{L}}, \end{array}$$

where *C*_*e*_ represents the equilibrium concentration of the metal ions (mg/L), *q*_*e*_ represents the amount of metal ions adsorbed by a unit mass adsorbent (mg/g), *q*_*L*_ represents the maximum amount of the metal ions adsorbed by the unit mass adsorbent (mg/g), and *K*_*L*_ represents the Langmuir constant (L/mg).

The Freundlich isotherm assumes multilayer sorption on a heterogeneous surface. The linear form of Freundlich equation can be expressed as [34]

(5) $$\begin{array}{c} \ln\left(\;q_{e}\;\right)=\ln\left(\;K_{F}\;\right)+\frac{1}{n}\ln\left(\;C_{e}\;\right), \end{array}$$

where *q*_*e*_ and *C*_*e*_ are the same as defined above and *K*_*F*_ and *n* are the Freundlich constants, which indicate the adsorption capacity and adsorption intensity of a given material, respectively. The *n* values between 1 and 10 indicate favorable adsorption [35].

**Figure 3:**
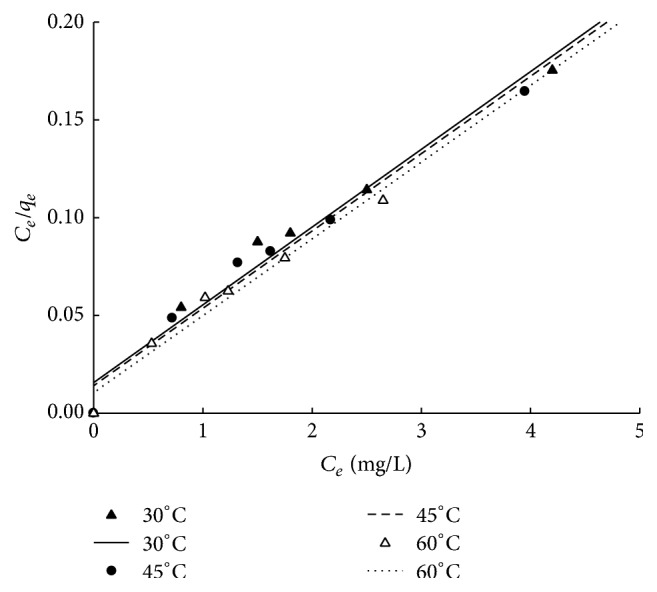
Langmuir model fit of modified biomass ash at different temperature.

Table 4 shows the fitting results of the parameters of the isotherm models at different temperatures. Both the adsorption models well fitted with the experimental data obtained for the natural biomass ash and the modified matrix. The Langmuir model (*R*^2^ = 0.963) showed a slightly better fit than the Freundlich model (*R*^2^ = 0.960), probably because the adsorption of Cd^2+^ by the biomass ash belongs to monolayer adsorption (Figure 3). With regard to the favorability of adsorption, the* n* values in the Freundlich model were greater than 3 at every temperature, indicating a favorable adsorption process of Cd^2+^ onto the modified biomass ash (Table 4). With the increase of temperature from 30°C to 60°C, the adsorption capacity of the modified biomass ash slightly increased. The possible explanation is that the adsorption process is an endothermic reaction, and the temperature can increase the internal structure of the modified biomass ash [36], thereby enhancing its adsorption capacity.

### 3.4. Kinetic Studies

**Figure 4:**
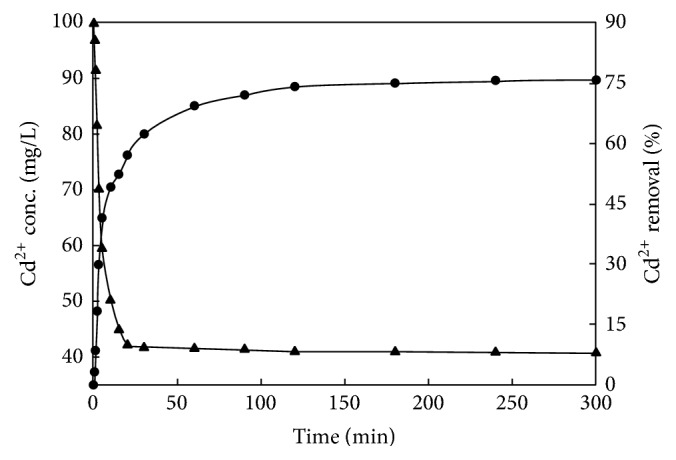
Sorption kinetics: concentration and removal (%) of Cd^2+^ from aqueous solution versus sorption time (initial concentrations of Cd^2+^, 100 mg/L; biomass ash concentration, 2 g/L; *T* = 30°C; initial pH 5.0).

The adsorption rate of the adsorbent in solution for metal ions can be understood by studying the kinetics of the adsorption process. Through the fitting of the experimental data with appropriate kinetic models, the adsorption mechanism can be understood.  Figure 4 shows the kinetics of Cd^2+^ sorption from aqueous solution onto the modified biomass ash (residual Cd^2+^ concentration versus contact time). The residual Cd^2+^ concentration was reduced sharply during the first 40–50 min and dropped to a value less than 40 mg/L within 120 min. At the initial stage of the reaction, the Cd^2+^ can be rapidly absorbed onto the adsorbent surface with a high number of active adsorption sites. As the adsorption proceeded, an increasing number of active adsorption sites are occupied by Cd^2+^, leading to a reduction in the sorption rate. The slower diffusion of Cd^2+^ onto the interior matrix of the modified biomass ash may also lead to the slower adsorption at the late stage [37].

In order to analyze the adsorption rate of Cd^2+^ on the modified biomass ash, we filled the results of the dynamic analysis to the pseudo-first-order and pseudo-second-order rate equations [38]. The two equations can be expressed as follows:

(6) ln⁡Qe−Qt=ln⁡Qe−k1ttQt=1k2Qe2+tQe,

where *Q*_*e*_ is the adsorption capacity (mg/g) at equilibrium, *Q*_*t*_ is the amount (mg/g) of material adsorbed at time *t*, *k*_1_ represents the rate constant (min^−1^) of the pseudo-first-order model, and *k*_2_ is the rate constant (g·mg^−1^·min^−1^) of the pseudo-second-order model.

**Figure 5:**
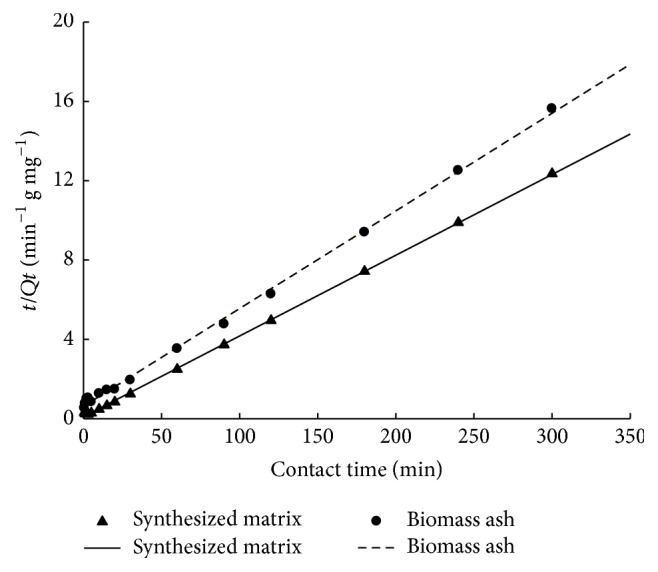
Plots of the pseudo-second-order kinetics for the adsorptions of Cd^2+^ on biomass ash and the synthesized matrix (initial concentrations of Cd^2+^, 100 mg/L; biomass ash concentration, 2 g/L; *T* = 30°C; pH 5.0).

Table 5 shows the kinetic parameters of the adsorption for Cd^2+^ on the biomass ash at 30°C. For the pseudo-second-order model, the *R*^2^ values were higher than those obtained using the pseudo-first-order model, especially for the modified biomass ash. The pseudo-second-order kinetic equations of the natural and modified biomass ash were higher than 0.99 (Figure 5), suggesting that the adsorption of Cd^2+^ followed the pseudo-second-order model and that Cd^2+^ ions were adsorbed onto the surface of both materials by chemical adsorption. The chemical adsorption may be caused by the reaction force and the coordination process between the Cd^2+^ and N–H groups (–NH_2_ and –NH–) on the surface of the modified biomass ash.

### 3.5. Thermodynamic Studies

Thermodynamic parameters including the standard Gibbs free energy (Δ*G*^0^), the standard enthalpy (Δ*H*^0^), and the standard entropy (Δ*S*^0^) for adsorption of Cd^2+^ onto the modified biomass ash were calculated using the following equations [39]:

(7) ΔG0=−RTln⁡KL′ln⁡KL2KL1=−ΔH0RT1−T2T2T1ΔS0=ΔH0−ΔG0T,

where *K*_*L*_′, *K*_*L*1_, and *K*_*L*2_ are the Langmuir constants at *T*, *T*_1_, and *T*_2_, respectively; *R* is the gas constant (8.314 J·mol^−1^·K^−1^).

Δ*G*^0^ is the adsorption driving force, which depends on Δ*H*^0^ and Δ*S*^0^.  Table 6 shows the thermodynamic parameters of Cd^2+^ adsorption on the modified biomass ash at different temperatures. The negative values of Δ*G* were indicative of favorable and spontaneous adsorption process. With the increase of temperature, the Δ*G* value was gradually decreased which showed that the increase of temperature was favorable to the adsorption process. The positive values of Δ*S* reflected the affinity of the modified biomass ash for Cd^2+^, while the positive value of Δ*H* indicated the adsorption was endothermic.

Moreover, adsorption can be divided into chemical adsorption and physical adsorption, both of which can occur simultaneously in one adsorption process [40]. The adsorption heat for van Edward force, hydrogen bond, ligand exchange, dipole interaction, and chemical bond is 4–10, 2–40, ≈40, 2–29, and >60 kJ·mol^−1^, respectively [41]. In our study, the Δ*H* value was 39.35 kJ/mol, indicating the adsorption by hydrogen bond and ligand exchange. Therefore, the adsorption of Cd^2+^ onto the modified biomass ash involved both physical and chemical adsorption.

## 4. Conclusions

A low-cost and efficient adsorbent was modified from biomass ash for the removal of Cd^2+^ from aqueous solution. Compared with the natural biomass ash, the modified material showed a much larger specific surface area and a greater number of active adsorption sites after modification with HMS and functionalization with APS. The modified product showed a better adsorption capacity for Cd^2+^ that was mainly dependent on the initial metal concentration and pH. The adsorption of Cd^2+^ on the modified biomass ash was well fitted to the Langmuir model. The adsorption processes were endothermic and followed the pseudo-second-order kinetics. Notably, the modified biomass ash showed a substantially higher adsorption capacity compared with the natural biomass ash as well as several types of adsorbent reported previously. This study provides a feasible method for utilizing biomass ash in the environment. Further investigation is needed to test the adsorption capacity of the modified biomass ash for other metal species from water.

## Figures and Tables

**Table 1:** Elemental composition of biomass ash analyzed by ICP-OES.

Element	Si	Ca	K	Al	Fe	Mg	Na	P	S	Mn	Ni	Zn	Cu	Cr	Pb	Cd
Proportion (%)	12.04	4.31	3.31	2.11	1.08	0.65	0.42	0.41	0.073	0.034	0.018	0.015	0.0035	0.0022	0.0005	0.0002

**Table 2:** Comparison of the BET analysis of functionalized hexagonal mesoporous silica, biomass ash, and synthesized matrix.

Analysis	Sample		
	HMS-NH2 [42]	Fly ash	Fly ash + HMS + 10% APS
BET surface area (m^2^/g)	17	21.38 ± 0.17	185.81 ± 0.15

**Table 3:** Comparison of Cd^2+^ adsorption capacities of different absorbents.

Absorbent	Adsorption capacity (mg/g)	pH	Reference
Synthesized matrix	25.00	5	
Peanut shell	0.93	5	[22]
Banana peel	5.71	5	[43]
Biochar	28.1	5	[44]
Coal fly ash	11.43	5	[45]
Bentonite	13.5	5.5	[46]
Sepiolite	8.11	5	[47]
Zeolite	6.72	6.5	[48]
Activated carbon	20.36	6	[49]

**Table 4:** Values of the constants and fitting of the adjusted adsorption models.

Adsorbent	Temp (°C)	Langmuir			Freundlich		
		*q*_*L*_ (mg/g)	*K*_*L*_ (L/mg)	*R* ^2^	*n*	*K*_*F*_ (mg/g) (mg/L)^1/*n*^	*R* ^2^
Biomass ash	30	20.83	1.37	0.996	6.85	13.53	0.976
	45	21.73	1.44	0.995	6.54	14.25	0.982
	60	22.22	1.67	0.994	6.45	14.57	0.986

Modified biomass ash	30	25.00	2.50	0.970	3.03	15.94	0.960
	45	25.64	3.55	0.974	3.34	16.48	0.953
	60	25.67	6.94	0.976	3.12	18.01	0.967

**Table 5:** Kinetic parameters for biomass ash and the synthesized matrix.

Adsorbent	Temp (°C)	Pseudo-first-order model		Pseudo-second-order model	
		*k*_1_ (min^−1^)	*R* ^2^	*k*_2_ (g·mg^−1^·min^−1^)	*R* ^2^
Biomass ash	30	8.8 × 10^−3^	0.724	3.71 × 10^−3^	0.999
Synthesized matrix		9 × 10^−3^	0.433	1.60 × 10^−2^	1

**Table 6:** Thermodynamic parameters for the synthesized matrix.

Temp (°C)	Thermodynamic parameters		
	Δ*G*^0^ (kJ·mol^−1^)	Δ*H*^0^ (kJ·mol^−1^)	Δ*S*^0^ (J·mol^−1^·K^−1^)
30	−2.31	39.35	137.49
45	−3.35		
60	−5.63		
